# *Legionella* spp. Risk Assessment in Recreational and Garden Areas of Hotels

**DOI:** 10.3390/ijerph15040598

**Published:** 2018-03-26

**Authors:** Antonios Papadakis, Dimosthenis Chochlakis, Vassilios Sandalakis, Maria Keramarou, Yannis Tselentis, Anna Psaroulaki

**Affiliations:** 1 Department of Clinical Microbiology and Microbial Pathogenesis, School of Medicine, University of Crete, Voutes—Staurakia, 71110 Heraklion, Crete, Greece; medp2011758@med.uoc.gr (A.P.); d.chochlakis@uoc.gr (D.C.); v.sandalakis@uoc.gr (V.S.); keramarou@gmail.com (M.K.); tselendi@med.uoc.gr (Y.T.); 2 Public Health Authority of Heraklion, 71201 Heraklion, Crete, Greece; 3 Regional Laboratory of Public Health, School of Medicine, 71110 Heraklion, Crete, Greece

**Keywords:** *Legionella*, recreational water systems, risk, water safety plan, hotel

## Abstract

Several Travel-associated Legionnaires’ disease (TALD) cases occur annually in Europe. Except from the most obvious sites (cooling towers and hot water systems), infections can also be associated with recreational, water feature, and garden areas of hotels. This argument is of great interest to better comprehend the colonization and to calculate the risk to human health of these sites. From July 2000–November 2017, the public health authorities of the Island of Crete (Greece) inspected 119 hotels associated with TALD, as reported through the European Legionnaires’ Disease Surveillance Network. Five hundred and eighteen samples were collected from decorative fountain ponds, showers near pools and spas, swimming pools, spa pools, garden sprinklers, drip irrigation systems (reclaimed water) and soil. Of those, 67 (12.93%), originating from 43 (35.83%) hotels, tested positive for *Legionella* (*Legionella pneumophila* serogroups 1, 2, 3, 6, 7, 8, 13, 14, 15 and non-pneumophila species (*L. anisa*, *L. erythra*, *L. taurinensis*, *L. birminghamensis*, *L. rubrilucens*). A Relative Risk (R.R.) > 1 (*p* < 0.0001) was calculated for chlorine concentrations of less than 0.2 mg/L (R.R.: 54.78), star classification (<4) (R.R.: 4.75) and absence of Water Safety Plan implementation (R.R.: 3.96). High risk (≥10^4^ CFU/L) was estimated for pool showers (16.42%), garden sprinklers (7.46%) and pool water (5.97%).

## 1. Introduction

*Legionella* bacteria live naturally in fresh water, as well as in artificial water systems such as hot water tanks, hot tubs or spas, cooling towers, plumbing systems, and decorative pools or fountains [1,2]. Hotel gardens are, also, frequently irrigated with sprinklers and these may present an additional risk, particularly if they utilize recycled grey-water or sewage-based water [3]. *Legionella* species are able to reproduce at 25–43 °C and able to survive at temperatures of up to 55–60 °C, making it possible for them to thrive even in hot water systems [4].

Two forms of legionellosis are caused by the *Legionella* pathogens: Legionnaires’ disease (LD), presenting with pneumonia-like symptoms, and Pontiac fever, presenting with influenza-like symptoms [5]. Legionnaires’ disease, a serious form of pneumonia, may be caused by any type of *Legionella* bacteria, although *L. pneumophila* serogroup 1 is considered the most virulent of all species and serogroups, causing approximately 75% of all *Legionella* infections [2,6,7]. Until now, more than 52 different species of *Legionella* with at least 73 different serogroups have been described, of which approximately 20 species have been associated with human disease [8,9,10,11]. The case-fatality ratio of LD has been recorded in the order of 10–15%. Usually the incubation period ranges from two to 10 days, but in rare cases it may be longer, for up to 16–20 days after exposure [12,13]. Since LD is normally acquired through the respiratory system by inhaling air that contains *Legionella* bacteria in an aerosol, droplets with a diameter of less than 5 μm (~90% showers aerosols) may come into contact with the lower human airways [8,14,15,16].

Travel-associated Legionnaires’ disease (TALD) corresponds to the cases of travelers who get infected in the country they visit, but usually get diagnosed and/or report their infection back in the country of their residence. These human cases do not include domestic ones, that is, cases in which humans travel within their own country. Due to the long incubation period (2–10 days), travelers may be exposed to *Legionella* bacteria in one country but develop symptoms and seek medical attention in another (such as their home) country [17]. According to the 2015 report by the European Centre for Disease Prevention and Control (ECDC), a total of 1141 human cases related to TALD were reported during that year at the 28 EU Member States and Norway, with the number being 20% higher than that in 2014; in 2014, a total of 953 human cases were reported (21% higher than the corresponding ones of 2013) [3]. The average risk to TALD ranged from 0.02 cases/million nights in the United Kingdom to 0.88 cases/million nights in Greece, according to a study carried out in 2009. In Greece, the pooled risk of 1.68 cases/million nights when travelling to the country was the highest among the 10 European countries [18,19].

Since 2004, the World Health Organization (WHO) has developed a Water Safety Plan (WSP) approach according to its Guidelines for Drinking Water Quality, which is based on risk assessment and risk management principles [20]. Together with the standard EN 15975-2 (concerning security of drinking water supply), these guidelines are an internationally recognized principle on which the production, distribution, monitoring and analysis of parameters in drinking water is based upon. In Europe, the Commission Directive Council 98/83/EC (EU) and 2015/1787 align with the principles and needs of the quality of water intended for human consumption. The WSP approach includes, also, supporting programs such as verification monitoring, appropriate documentation and record-keeping, training and communication [21,22].

The aims of the present study were: (1) culture and identify *L. pneumophila* and *Legionella* species in environmental samples obtained from recreational areas and determine the frequency and severity of colonization of *Legionella* in these sites; (2) estimate the risk factors associated with *Legionella* colonization of recreational systems and (3) evaluate the implementation of WSPs to limit *Legionella* colonization.

## 2. Materials and Methods 

### 2.1. Inspections—Sample Collection

During the period of 2000–2017, the Local Public Health Authorities of the Island of Crete in Greece inspected 119 hotels associated with TALD. Environmental samples from decorative fountain ponds, showers near pools and spas, swimming pools, spa pools, garden sprinklers, drip irrigation systems (reclaimed water) and soil were collected from each hotel, where applicable. 

Specifically, as regards the reclaimed water from the drip irrigation systems, in all sample cases it consisted of a product of secondary biological treatment followed by advanced treatment and disinfection. According to the National legislation “Common Ministerial Decision (CMD) No 145116: Measures, limits and procedures for reuse of treated wastewater (Issuing Institutions: Ministry of Environment, Energy and Climate Change)”, this kind of water can be used unrestrictedly in irrigation of gardens and in recreational areas of hotels provided that it fulfills the following criteria: (1) Total coliforms (TC) (cfu/100 mL) ≤2 in 80% of samples and ≤20 in 95% of samples; (2) Biochemical oxygen demand (BOD_5_) (mg/L) ≤10 in 80% of samples; (3) Total suspended solids (TSS) (mg/L) ≤2 in 80% of samples; (4) Turbidity (NTU) ≤2 median. The above criteria are met following secondary biological treatment followed by advanced treatment and disinfection.

The sample sites were chosen based on the sites that could produce aerosols and on the sites (rooms, areas and so on) potentially associated with a TALD case. The samples were collected according to: (a) the guidelines for drinking-water quality (second edition) and (b) ISO 5667-2:1982—Part 2: guidance on sampling techniques; since 2006, samples were collected following the ISO 19458:2006 Water quality—Sampling for microbiological analysis methodology. The samples were labeled and temporarily stored in a cool box at a temperature of up to 5 (±3) °C protected from direct light, before being delivered to the laboratory immediately after the sampling (no more than 24 h).

### 2.2. Data Collection

Data on water temperature, pH, chlorine concentration, disinfection methodology, hotel star rating, number of rooms/beds, implementation of a WSP, season and water supply were recorded. Inspections were conducted following a checklist developed with some information such as name, address of building, type of hot water production system, water disinfection system, periodicity and type of water system maintenance and cleaning, water supplying and number of rooms and beds. The temperatures were measured (two minutes after flushing) using a calibrated thermometer, placed in the middle of the water stream. Free chlorine and pH were measured using a calibrated portable, microprocessor-based meter. Samples were collected in 1 L sterile containers containing sufficient sodium thiosulphate (20 mg) to neutralize any chlorine or other oxidizing biocides.

### 2.3. Plate Culture Method

The isolation of *Legionella* from water samples was performed by culture according to the International Standard method ISO 11731 (1998) and ISO 11731-2 (2004). The latter ISO was implemented from 2004 onwards. Briefly, water samples were concentrated by filtration and were re-suspended in Distilled Deionized water. A volume of the suspension (200 μL) was spread on BCYE (Buffered Charcoal Yeast Extract), BCY (Buffered Charcoal Yeast Extract without l-cysteine) and GVPC (Glycine Vancomycin Polymyxin Cycloheximide) (Biomérieux, Craponne, France) Petri dishes: (a) directly after filtration; (b) after incubation at 50 °C for 30 min and (c) after the addition of an acid buffer (0.2 mol/L solution of HCL, pH 2.2). The detection limit of the procedure was 50 CFU/L. The inoculated plates were incubated for 10 days at 36 ± 1 °C in 2.5% CO_2_ with increased humidity. Suspected colonies were randomly chosen for subculture on BCY, BCYE and GVPC agar.

### 2.4. Typing of Legionella Isolates

Up until 2010, the isolated colonies were identified using an agglutination test (SLIDEX *Legionella*-Kit, Biomérieux, Craponne, France), which allows for the discrimination of *L. pneumophila* serogroup 1 from serogroups 2–14 and of *L. anisa*, while for the exact detection of each *L. pneumophila* serogroup, individual latex polyclonal reagents were used (Pro-lab, Richmond Hill, ON, Canada).

### 2.5. Identification—MALDI-TOF Mass Spectrometry

From 2010 onwards, a MALDI Biotyper (Microflex LT MALDI-TOF mass spectrometer) (Bruker Daltonics, Leipzig, Germany) equipped with a microSCOUT ion source was used for the identification of individual *Legionella* colonies against its microbial database (v 3.1.2.0). Spectra were recorded using the flexControl software with the default parameters for optimization set by the manufacturer (Bruker Daltonics, Leipzig, Germany). For each spectrum, 240 laser shots were collected and analyzed (6 × 40 laser shots from 120 different positions of the target spot). All identifications were evaluated according to the manufacturer scoring scheme.

### 2.6. Risk Assessment of Legionella Presence in Water Distribution Systems

To better calculate the risk, we used two different approaches. The first one was in accordance with the recommendations of the European Legionnaires’ disease Surveillance Network (ELDSNet), at which the microbiological results of the water samples were analytically and statistically analyzed according to the number of *Legionella* bacteria in the water sample, which could represent a particular risk to the human health. In particular, an insignificant risk was noted at ≤10^3^ CFU/L, medium risk at >10^3^ CFU/L but <10^4^ CFU/L, and high risk at ≥10^4^ CFU/L. Especially for spa pools, the recommendations point to the risk of legionellosis following *Legionella* sampling to low risk (>10^2^ but <10^3^) and to high risk (≥10^3^ CFU/L) [23]. A second inspection was performed at a hotel when the *Legionella* count was ≥10^4^ CFU/L in at least one sample or between >10^3^ and ≤10^4^ CFU/L in more than two samples or between >10^3^ and ≤10^4^ CFU/L in at least one sample in concordance with an aerobic count higher than 10^5^ CFU/L.

The second approach for the assessment of the risk was based on a semi-quantitative risk matrix approach, which was used to implement the water supply system risk assessment (Table 1). The risk levels were divided into: high (≥20), medium (10–19), and low (<10). The risk (R) was evaluated based on the following parameters: Likelihood (L) or the occurrence of accidents/damage, frequency of the risk exposure, consequence or severity (S). The level of the risk was calculated as follows: R = L × S [24,25].

### 2.7. Instructions Given in Case of Positive Samples

According to the national guidelines, in samples which met the criteria of >10^3^ to <10^4^, either: (i) if a small proportion of samples (10–20%) were positive, the system was re-sampled. If a similar count was recorded again, then a review of the control measures and risk assessment was carried out to identify any remedial actions; (ii) if the majority of samples were positive, the system was considered as colonized, even at a low level, with *Legionella*. Disinfection of the system was considered but an immediate review of control measures and a risk assessment was carried out to identify any other remedial actions required. In samples which met the criteria of ≥10^4^: The system was re-sampled and an immediate review of the control measures and risk assessment was carried out to identify any remedial actions, including whether a disinfection of the whole system or affected area was necessary.

### 2.8. Implementation of a Water Safety Plan

As from 2005, a detailed standardized questionnaire (checklist, Appendix A) was used to evaluate the risk associated with the non-implementation of a WSP. The checklist consisted of 42 scoring items (11 of which were designated as “critical”), which were classified into seven categories: construction and maintenance; cleaning and disinfection; cold-water distribution system; hot-water distribution system; system protection cross-connections and backflow; record keeping; and on-site manually conducted tests. The total negative score was calculated and classified qualitatively in the following three categories: satisfactory result (0–7 points, <10% of the total negative score, no critical violation), relatively satisfactory result (8–14 points, 11–20% of the total negative score, or a critical violation), and unsatisfactory result (more than 14 points, >20% of the total negative score).

### 2.9. Statistical Analysis

All statistical analyses were conducted using the IBM SPSS Statistics Version 24 statistical package, the Epi-Info 2000 version 7.2.0.1 (Centers for Disease Control and Prevention, Atlanta, GA, USA) and the MedCalc relative risk calculator statistical software free online version; Relative risk (R.R.) at a 95% confidence interval (CI). The analyses were calculated to assess categorical risk variables from water distribution systems and hotel characteristics, associated with *Legionellae*-positive test results. The results were considered statistically significant when the *p* value was <0.05 and highly significant when the *p* value was <0.0001.

## 3. Results

### 3.1. Descriptive Data

Of the 518 samples collected, 67 (12.93%) originated from 43 (35.83%) hotels that tested positive for *Legionella* species. The mean positivity was 9.41% (standard deviation of 11.91, max of 33.33% min 0.00%) (Table 2). A second inspection was required for 49 hotels, a third for 16 hotels, a fourth for five (5) hotels, while a fifth inspection was required for two (2) hotels. The repetitive inspections were carried out in cases where the implementation of measures did not deliver the expected results in terms of the presence of *Legionella* species at the sites tested and/or the risk factors (as these are explained below) were repeatedly tested out of the limits.

In 14/119 (28.57%) of the hotels inspected, a cluster was noted (clusters were defined as the presence of two or more cases having stayed overnight at the same accommodation site in the 14 days before onset of illness and whose illness was within say the same two-year period) [23]. 

### 3.2. Isolation and Identification of L. pneumophila and Legionella Species in Environmental Samples Obtained from Recreational Systems

*Legionella* was isolated from swimming pool showers, garden sprinklers, reclaimed water, swimming pool water, decorative fountain, spa water and spa showers. On the contrary, no *Legionella* species were isolated from shower heads, from Jacuzzis and from garden soil. All results are summarized in Table 2.

*Legionella pneumophila* serogroups 1, 2, 3, 6, 7, 8, 13, 14, 15 and 2–15 were detected. Of the non-pneumophila species, *L. anisa*, *L. erythra*, *L. taurinensis*, *L. birminghamensis*, *L. rubrilucens* and *Legionella* species were identified, the majority of which, belonged to the human pathogenic species *L. anisa*. The CFU/L ranged from 50–350,000. The lowest CFU/L were detected in spa waters, while the highest ones were identified in swimming pool showers and in garden sprinklers. All results are summarized in Table 3.

### 3.3. Univariate Examination of Risk Factors

The presence of *Legionella* was possibly associated (statistically significant correlation together with a relative risk of >1) with: (a) the implementation of a WSP; (b) free chlorine <0.2 mg/L; (c) hotel star classification of <4; (d) seasonal operation; (e) municipality hosting hotels population of <10^4^ people and (f) absence of an automated chlorination system together with free chlorine out of range. The number of beds of >200, the number of rooms of >80 and the use of groundwater as a source of water supply showed a statistically significant correlation in the absence of a relative risk >1. On the other hand, no statistically significant correlation was calculated for cold water temperature of >25 °C, temperature of >20 °C, the absence of an automated chlorination system, pH out of limits value (pH within limits 7.0–7.8), the opening/closing period and the high season period (June–August). All results are summarized in Table 4.

**Figure 1:**
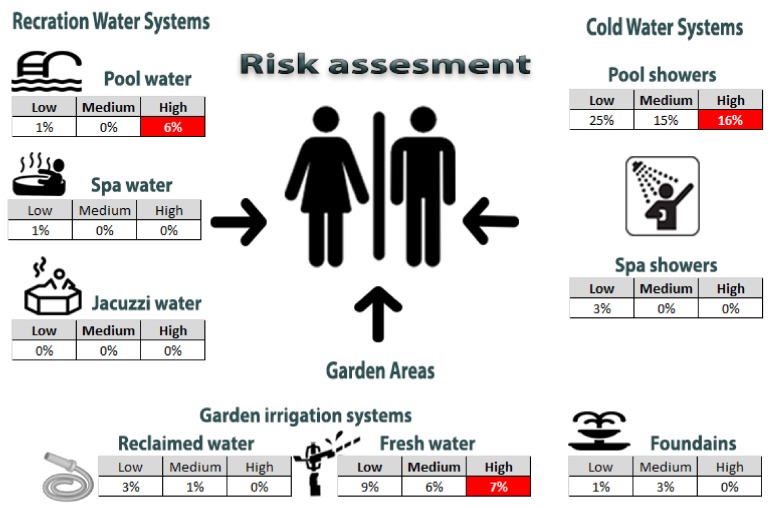
Risk assessment for *Legionella* contamination based on the CFU/L detected in each site tested.

Based on the risk assessment according to the guidelines of ECDC (low, medium and high for CFU/L < 10^3^, 10^3^–10^3^ and >10^4^ respectively) a corresponding Figure 1 was built to demonstrate the risk for each site tested. According to the semi-quantitative risk assessment a number of areas were designated as low, medium and high according to the likelihood of any species been present, the severity caused in the presence of any *Legionella* and the total risk (as a factor taking these two parameters into consideration). Of the total sites tested, the garden sprinklers and the swimming pool showers presented a higher risk when all three hazardous events were considered. As regards the pool water, a high risk was calculated for the event of finding *Legionella* in the water system, only. All results are summarized in Table 5.

### 3.4. Evaluation of the Implementation of WSPs

Data from the use of the checklist were collected from 51 hotels. The major recordings had to do with water storage tank protection, clean showers, presence of the proper concentration of residual chlorine, and water temperatures. All findings are summarized into Table 6.

Of the 51 hotels for which the questionnaire for the correct implementation of a WSP was filled in, 24 (36.4%) received a score B (relatively satisfactory) while 37 (63.7%) got a score C (unsatisfactory).

A higher colonization was recorded in the absence of a WSP (R.R. 3.04; *p* value <0.0001; CL 95% 1.73 to 5.34; *z*-statistic 3.87; NNT (Harm) 8.06) and in hotels where wrong implementation of WSP was recorded (R.R. 3.78; *p* value 0.0077; CL 95% 1.42 to 10.08; *z*-statistic 2.66; NNT (Harm) 7.33).

## 4. Discussion

*Legionella* is a naturally occurring microorganism found in freshwater environments and surface waters where it exploits free-living amoeba cells for survival and multiplication, and, in that context, it is not hard to be introduced in building water systems through the main community water supply network. In fact, it has been stated that in the USA a percentage of 87.5% of Legionellosis outbreaks has been related to community water systems [26]. Even though the colonization of a water system by *Legionella* occurs frequently, this alone is not enough to pose a high risk to humans, unless the bacteria population reaches high numbers and becomes dispersed through appropriate aerosolization. For such conditions to take place, a series of variables must co-occur, such as the increased temperature of cold water or decreased temperature of hot water (optimally 35–46 °C), absence of water disinfectants or under-treated water, out of range pH values (5.5–9.2), presence of substances like iron salts and l-cysteine, as well as, co-existing and supporting microbial flora. Furthermore, other factors such as the complexity of the architecture of the pipe system (high surface to volume ratio), presence of blunt-end pipes, infrequently used pipe terminals, pipelines exposed to the sun heat, close proximity of hot and cold-water pipelines, insufficient maintenance and end-points creating aerosols, have all been evaluated to significantly affect the rate and extent of *Legionella* colonization in water systems [27]. All these factors may well be associated with the formation of biofilms, which can provide a means for the survival and dissemination of the pathogen and undermine the efforts to eradicate bacteria from water systems [28].

The presence of 1559 hotels and accommodation units in Crete with 85,407 rooms and 161,578 beds (data drawn from the Hellenic National Service of Tourism for 2015), could not exclude the island from the presence of TALDs [29]. The current research was focused mostly on the significance of sampling from recreation areas and spas and in particular from pools, spas, jacuzzis, showers next to swimming pools, shower heads and spa showers. The calculated relative risk of *Legionella* colonization was less in swimming pools and in spas, due to the increased surveillance and implementation of proper chlorination procedures, as opposed to the colonization in showers of swimming pools. The analyzed data showed that the high chlorination, the mixed type of contamination and the lower temperatures in the pool are potential factors that may contribute to the prevention of any *Legionella* from reproducing and reaching levels that would represent a health risk for bathers. The pool samples from which *Legionella* were isolated, had significantly lower concentrations of residual free chlorine, in fact less than the 0.4 mg/L required by the current regulations in Greece (0.4–0.7 mg/L). On the other hand, no significant correlation between *Legionella* colonization and pH values was calculated in the current study, agreeing with the study by Fragou et al., where the colonization of *Legionella* in 116 samples collected from hotels and hospitals was evaluated against pH values [30]. In general, such conditions support the growth of *Legionella*, while the frequent vigorous agitation that can occur in spas, pools and whirlpool baths can form aerosols that could create airborne bacteria and transfer them to the lungs of unsuspected individuals [31,32], even if the actual establishments are not actively used by these humans [33]. This is, also, supported by the detection of *Legionella* even in more isolated water facilities such as in spa and pools located on cruise ships that are actually one of the most common sources of *Legionella* infection [32]. It seems that such aerosols-creating settings, along with the close proximity of susceptible individuals, create a potential *Legionella* infectious environment. Such conditions are recorded in showers, especially the ones that have their pipes or at least part of them exposed to the sun heat. Similar results have been described from researches in Italy [4] a country that, like Greece, is a major tourist destination and retains a Mediterranean climate. In fact it has been shown that the risk for travelers is high in southeastern countries (including Greece) compared to countries with moderate climates [19]. Hotel fountains creating aerosols certainly mimic the conditions met in showers and in most cases they use recirculating water that is under a minimal degree of disinfection supervision, if any at all. Therefore, they have already been linked to Legionellosis outbreaks since the bacterium finds it easy to thrive in such conditions and disperse through the formed aerosols to the surrounding environment, or at a much larger distance if favoring conditions exist (for example the presence of wind) [34].

In our study, positive samples were recorded in 43/119 (36.13%) hotels, a result comparable to a similar study in Turkey, where of the 52 hotels included in the study, negative samples for *Legionella* were retrieved in 16 (30.8%) hotels [35]. According to the results of the current study, the seasonal operation of hotels and their presence in municipalities with low population seems to play a role in the increased risk of *Legionella* colonization; this finding has already been supported in a previous study carried out in hotels in Greece [36].

Another point of interest was the high number of *Legionella* species isolated, indicating a widespread dispersion of these microorganisms in the environment. In addition to the isolation of *L. pneumophila* (serogroups 1, 2, 3, 6, 7, 8, 13, 14, 15, and 2–15), other potentially pathogenic environmental species were also isolated, such as *L. anisa*, *L. erythra*, *L. taurinensis*, *L. birminghamensis*, and *L. rubrilucens*, the agent responsible for Pittsburg pneumonia [37] which is similar to Pontiac fever [38]. The presence of non-pneumophila species should not be underestimated since human cases of infection by *Legionella* species other than *L. pneumophila* or even the second most common, *L. anisa*, have been described worldwide, including the Island of Crete [39].

*Legionella pneumophila* serogroup 1 was detected in swimming pools and swimming pool showers however the low number of samples collected did not allow us to make any correlation among the positive samples, the detected serogroups and the sampling sites. The highest CFU/L was detected for the *L. pneumophila* serogroup 14 (10^5^ CFU/L) and for *non-pneumophila* species (200,000 CFU/L). Nevertheless, a larger number of positive samples is required before coming to concrete conclusions on a possible relationship between site of collection and number of bacteria. The prevalence of *Legionella* species at certain environments has, also, been investigated by Litwin and colleagues [26] in which, they isolated *L. pneumophila* serogroup 1 and 6, *L. anisa, L. feeleii*, and *L. quateriensis* in the water reservoirs of recreational vehicles. As far as seasonality is concerned, in a similar research it was claimed that the concentrations of *Legionella* recovered from swimming pools, like *L. micdadei* and *L. bozemanii*, did not show any seasonal trend [4].

As regards the use of reclaimed water, ingestion of enteric pathogens causing gastrointestinal disease poses the greatest risk from exposure to wastewater however, inhalation of aerosols or dermal contact can, also, lead to disease. Except from *Vibrio cholerae*, the rest of the enteric pathogens that may cause gastrointestinal illnesses do not grow or survive for ever in water. On the other hand, free-living pathogens, such as *Legionella*, can grow under favorable conditions in treated wastewater and associated biofilms and, under certain circumstances, can survive within amoeba in water distribution systems [40]. That is why, samples from garden drip irrigation systems should be collected and tested in cases where reclaimed water is used and above all, it must be made clear that this type of water should not be used for irrigation through spraying [41].

Concerning WSPs, they already exist in various contexts worldwide. For instance, WSP networks are located in Africa, the Asia-Pacific region and in Latin America. WSPs have been designed in order to monitor water utility and to ensure that its quality standard is consistently kept within accepted limits [42]. In another study carried out in Germany, a decreased number of transgressions was observed following implementation of WSP in all parts of a hospital, supporting its efficacy [43]. In a different study carried out in Iceland, the impacts of WSPs on drinking water quality and health were evaluated and positive conclusions were drawn since implementation of WSPs resulted in better compliance in drinking water, reduced Heterotrophic Plate Count (HPC) numbers in water and decreased incidence of diarrhea in communities served by utilities implementing WSP [44]. It should be noticed however, that it turns out that it not just the presence of a WSP that will ensure the diminishing of the danger for a human infection by water-borne pathogens (including *Legionella*); its proper implementation is sometimes a more crucial factor [45]. In fact, it is possible that the improper implementation or the complete absence of a WSP (leading sometimes to temperature and pH values out of range) may provide the ideal stress conditions for the *Legionella* bacteria to develop a decreased sensitivity to antibiotics [46]. Of course, this is a huge aspect that needs further investigation.

Summing up, there is a need for awareness strategies in concordance with the operators of accommodation sites for the prevention of human *Legionella* infections. Stronger evidence on the source of infection can only be supported through enhanced standardization of *Legionella* investigation, reporting and follow-up, together with the use of high discrimination laboratory techniques. These factors could eventually lead to a more effective measure control [17]. Moreover, maintenance, regular controls, interventions on the hydraulic system, and good hygiene practices together with the assessment of risk analysis and establishment of a routine environmental microbiological surveillance schemes should be implemented to minimize exposure to *Legionella* infection [47,48].

## 5. Conclusions

The presence of *Legionella* is one of the most serious microbiological risks according to the water safety in distribution hotels. Knowing that *Legionella* bacteria can successfully thrive in natural and artificial water environments with warm waters, certainly directs towards the investigation of establishments with such conditions and with high human use or of those which present more complicated constructions such as pools and spas.

Preventing recreational water LD is a multifaceted issue that requires both the development of a WSP and, above all, its correct implementation, together with the participation of a large group of people like hotel staff and national and local public health authorities.

## Figures and Tables

**Table 1:** Presentation of the semi-quantitative risk matrix approach that was used to implement the water supply system risk assessment. The risk levels were divided into: high (≥20), medium (10–19), and low (<10). The risk (R) was evaluated based on the following parameters: Likelihood (L) or the occurrence of accidents/damage, frequency of the risk exposure, consequence or severity (S). The level of the risk was calculated as follows: R = L × S.

Severity or Consequence						
		Insignificant (Wholesome water) **Rating: 1**	Minor (Short term or localised, not health related non-compliance or aesthetic) **Rating: 2**	Moderate (Widespread aesthetic issues or long-term non-compliance, not heath related) **Rating: 4**	Major (Potential long-term health effects) **Rating: 8**	Catastrophic (Potential illness) **Rating: 16**
**Likelihood or** **F** **requency**	Most unlikely (Has not taken place in the past and it is highly improbable that it will occur in the future) **Rating: 1**	1	2	4	8	16
	Unlikely (Is possible and cannot be ruled out completely) **Rating: 2**	2	4	8	16	32
	Foreseeable (Is possible and under certain circumstances could occur) **Rating: 3**	3	6	12	24	48
	Very likely (Has occurred in the past and has the potential to occur again) **Rating: 4**	4	8	16	32	64
	Almost certain (Has occurred in the past and could occur again) **Rating: 5**	5	10	20	40	80

**Table 2:** Collection sites and sites where *Legionella* species were detected. The percentages of the positive samples have been calculated based on the total number of collected samples. The ranges have been assigned based on the European Centre for Disease Prevention and Control recommendations. The numbers at the left side of the slash (/) correspond to the sample findings and the numbers at the right side of the slash (/) correspond to the hotel findings.

Original Sample Description	Samples/Hotels		Range (CFU/L)		
	Total No	Positive	≤10^3^	>10^3^ and <10^4^	≥10^4^
Reclaimed Water	13/9	3 (23.08%)/2 (2.22%)	2 (66.67%)/2 (22,22%)	1 (33.33%)/1 (11,11%)	-
Decorative Fountains	45/24	3 (6.67%)/3 (12.50%)	1 (33.33%)/1 (4.17%)	2 (66.67%)/2 (8.33%)	-
Shower Heads	2/1	0	-	-	-
Garden Sprinklers	37/21	15 (40.54%)/11 (52.38%)	6 (40%)/6 (28.57%)	4 (26.67%)/4 (19.05%)	5 (33.33%)/5 (23.81%)
Jacuzzi Water	15/10	0	-	-	-
Pool Water	107/61	5 (4.67%)/4 (6.56%)	1 (20%)/1 (1.64%)	-	4 (80%)/3 (4.92%)
Spa Water	10/7	1 (10%)/1 (14.29%)	1/1 (100%)	-	-
Swimming pool Showers	271/104	38 (14.02%)/30 (28.85%)	17 (44.74%)/15 (50%)	10 (26.32%)/9 (30%)	11(28.95%)/10 (33.33%)
Spa Showers	16/7	2 (12.50%)/2 (28.57%)	2 (100%)/2 (28.57%)	-	-
Garden Soil	2/1	0	-	-	-
Total	518/119	67 (12.98%)/43 (36.13%)	30 (44.78%)/25 (21.01%)	17 (25.37%)/4 (11.76%)	20 (29.85%)/14 (11.76%)

**Table 3:** *Legionella* serogroups and species isolated and identified from recreational waters (Ranges as CFU/L).

*Legionella* Serogroups/Species	Swimming Pool Water		Spa		Swimming Pool Shower		Spa Shower		Fresh Water from Garden Sprinklers		Decoration Fountain		Reclaimed Water	
	Pos.	Range	Pos.	Range	Pos.	Range	Pos.	Range	Pos.	Range	Pos.	Range	Pos.	Range
*L.p.* sg 1	1	700			5	350–1150			1	26,000				
*L.p.* sg 2					4	100–2050								
*L.p.* sg 3									3	50-650				
*L.p.* sg 6					1	150	2	150–600						
*L.p.* sg 7					5	200–3350								
*L.p.* sg 8					2	50			1	300				
*L.p.* sg 13									1	32,500				
*L.p.* sg 14			1	50	3	150–100,000	1/1	150	3	250–13,000				
*L.p.* sg 15														
*L.p.* sg 2–15					8	50–100,000			4	13,500–200,000			1	1000
*L. anisa*	2	19,500–26,500			9	250–350,000			3	50–13,000	1	2500		
*L. erythra*			1	650	3	400–13,000			1	200	1	1500		
*L. taurinensis*					2	50			1	6500	1	2000		
*L. birminghamensis*					1	650–8250			1	65,000				
*L. rubrilucens*	1	26,000			3	50–6500								
*L. species*	1	200,000			4	50–1000					1	1000	3	1000

Pos.: positive. *L.p.*: *Legionella pneumophila*. sg: serogroup.

**Table 4:** Association of water distribution systems and hotel characteristics with *Legionella* colonization. The terminology number needed to treat (NNT) has been used as proposed at the free online relative risk calculator statistical software, MedCalc (Altman 1998). In our case, the term benefit is associated with the number of additional inputs required for each parameter tested to get a positive association with this parameter.

Risk Factors	R.R.	95% CI	z Statistic Statistic	*p* Value	NNT
Free chlorine <0.2 mg/L	54.78	20.47–148.04	7.94	<0.0001	(Harm) 1.85
No Water Safety Plan	3.96	2.32–6.75	5.04	<0.0001	(Harm) 6.06
Wrong implementation of WSP	3.78	1.42–10.08	2.66	0.0077	(Harm) 7.33
Number of rooms >80	0.29	0.17–0.47	4.83	<0.0001	(Benefit) 6.25
Number of beds >200	0.22	0.12–0.40	5.08	<0.0001	(Benefit) 5.89
Star classification <4	4.75	2.80–8.06	5.78	<0.0001	(Harm) 4.95
Groundwater as a source of water supply	0.27	0.13–0.58	3.34	0.0008	(Benefit) 5.70
No automated chlorination system and free chlorine <0.2 mg/L	5.16	2.58–10.31	4.65	<0.0001	(Harm) 3.60
pH out of limits and free chlorine <0.2 mg/L	4.52	1.71–11.96	3.04	0.0024	(Harm) 3.08
Population < 10,000	1.61	1.03–2.58	2.11	0.02	(Harm) 15.78
No automated chlorination system	1.93	0.71–5.25	1.29	0.04	(Harm) 12.57
Closing period	1.56	0.87–2.78	1.51	0.07	(Harm) 14.59
Seasonal operation	1.47	0.66–3.28	0.94	0.17	(Harm) 23.93
Cold water >25 °C	1.38	0.82–2.64	1.29	0.14	(Harm) 18.17
Cold water >20 °C	1.21	0.70–2.08	0.70	0.35	(Harm) 39.29
pH out of limits	0.68	0.2–1.98	0.69	0.25	(Benefit) 18.59
High season months	1.10	0.70–1.730	0.44	0.65	(Harm) 76.27
Opening period	1.03	0.35–3.05	0.06	0.45	(Harm) 214.76

**Table 5:** Risk assessment of the sites based on the likelihood for the presence of *Legionella* (*), the severity raised if *Legionella* is present (^) and the final risk score calculated (#). The final risk score was calculated based on the findings of the previous two parameters.

Area	Hazard and Hazardous Event	Likelihood or Frequency *	Severity or Consequence ^	Risk Score #	Risk Rating #
Reclaimed Water	Event of finding *Legionella* in the water system	3	4	12	Medium
	Inadequate disinfection method	3	4	12	Medium
	Low chlorine residual in distribution systems	3	4	12	Medium
Decorative Fountains	Event of finding *Legionella* in the water system	3	4	12	Medium
	Inadequate disinfection method	3	4	12	Medium
	Low chlorine residual in distribution systems	3	4	12	Medium
Shower Heads	Event of finding *Legionella* in the water system	2	4	8	Low
Garden Sprinklers	Event of finding *Legionella* in the water system	5	8	40	High
	Inadequate disinfection method	5	8	40	High
	Low chlorine residual in distribution systems	5	8	40	High
Jacuzzis Water	Event of finding *Legionella* in the water system	2	4	8	Low
	Inadequate disinfection method	2	4	8	Low
	Low chlorine residual in distribution systems	2	4	8	Low
Pool Water	Event of finding *Legionella* in the water system	3	8	24	High
	Inadequate disinfection method	2	8	16	Medium
	Low chlorine residual in distribution systems	2	8	16	Medium
Spa Water	Event of finding *Legionella* in the water system	2	4	8	Low
	Inadequate disinfection method	2	4	8	Low
	Low chlorine residual in distribution systems	2	4	8	Low
Swimming pool Showers	Event of finding *Legionella* in the water system	4	8	32	High
	Inadequate disinfection method	4	8	32	High
	Low chlorine residual in distribution systems	4	8	32	High
Spa Showers	Event of finding *Legionella* in the water system	2	4	8	Low
	Inadequate disinfection method	1	4	4	Low
	Low chlorine residual in distribution systems	1	4	4	Low
Garden Soil	Event of finding *Legionella* in the water system	1	4	4	Low

**Table 6:** Findings from the completion of the checklists at the 51 hotels. Only the items, for which a deviation from the normal value was recorded and are presented herein.

Scoring Items	%
No water storage tanks protection	100
* The showers are NOT clean and free of salts	81.2
* The residual chlorine <0.2 mg/L	72.8
The hot water temperature is <50 °C. after 1 min of flowing	63.6
The cold-water temperature in taps is >25 °C. after 2 min of flushing	54.5
Water store and circulation temp <60 °C	54.5
The water exiting the heating unit <60 °C and returning <50 °C	54.5
The amount of stored water is >1 day	45.5
There is NO control book	45.5
* The outgoing temperature (cold) water from the tank is >25 °C	36.3
There are leaks in the network	27.2
* NO random checks of water at least every 6 months	27.2
There is no thermal stratification of the water inside the heaters and storage water	27.2
The water distribution system cannot provide adequate water supply at peak times	18.2
There is a change (increase or decrease) in the consumption of the water	18.2
* Detected *Legionella* the last six months (at a concentration of more than 10^3^ CFU/L)	18.2
The filters are NOT in good condition	9
* The network is NOT cleaned and disinfected when remained out of service >1 month	9
* The network and the tanks NOT cleaned with appropriate disinfectants at least annually	9
The water supply is interrupted for a long time	9
* The difference in temperature between 2 successive measurements of hot water is >10 °C/1 min	9
There is a taste and odor problem	9

*: designates a critical item.

## Appendix A

**Table A1:** Detailed standardized questionnaire (checklist) used to evaluate risk associated with proper or not implementation of a WSP. * Critical Control Point. The checklist has been adapted from the European Guidelines for Prevention and Control of Travel Associated Legionnaires’ disease and from the National School of Public Health 2004 Athens Olympic Games Checklist for building water systems.

*Legionella* Prevention and Management Checklist				
Item to Check		Yes	No	Remarks
A water safety plan is implemented according to the requirements of the European Legislation and the proposals of the World Health Organization				
Is there at least one named person responsible for *Legionella* control?				
Responsible Person: (entity name)				
Is this person properly trained in the control of Legionella and able to understand the system(s), risk factors and control measures?				
Is there a written *Legionella* control plan in place for each system which could pose a risk?				
If the hotel is operating on a seasonal level, is the entire domestic water system disinfected with high level (50 mg/L) chlorine for 2–4 h before the property is reopened?				
Has there been any new pipe work or changes to existing pipe work in the last two years?				
If yes, has it been checked to ensure that there are no pipes with intermittent or no water flow?				
Was the system disinfected with high level chlorine or heat (60 °C) following completion of the work?				
**General Testing Sites of the Water Network**				
1	The pressure on the meter is 1–12 atmospheres		−1	
2	The filters are in good condition		−2	
3	The sealing is in good condition		−2	
4	Absence of leaks on the network		−2	
5 *	The storage tank is maintained in good condition and no sediments are observed inside it		−3	
6	The water storage tanks have lids and wire mesh in each open air duct		−1	
7	The amount of water stored is no greater than one day’s use		−1	
8 *	The network is cleaned and disinfected when it is not under use for more than a month		−3	
9 *	The network and the tanks are cleaned with appropriate disinfectants at least once a year		−3	
10	The water supply is not interrupted for long periods of time		−1	
11	Unused taps are removed from the network		−2	
12	Checking water network diagrams			
**Cold Water Systems**				
13	Chillers are maintained in good condition		−1	
14	Chiller filters are maintained in good condition		−1	
**Hot Water Systems**				
15	The system responds well at peak hours		−1	
16	There is no change (increase or decrease) in water consumption		−1	
17 *	No standing water in piping for more than one week		−3	
18 *	If No: Is a flushing procedure used?		−3	
19 *	Shower heads and taps are clean and free from scale		−3	
**Water Heating and Storage Appliances**				
20	The appliance is dried and checked		−1	
21	The appliance is cleaned if necessary		−2	
22	The hot water extraction duct is dried		−1	
23	The appliance is maintained in accepted sanitary condition		−2	
**Faucets**				
24	Operated and maintained in accordance with the manufacturing instructions		−2	
**Fire Protection Water Supply**				
25	No backflow of water fire extinguishing system in the water supply network		−2	
**ANNEX Ι: Database Book**				
26	Are there regular records (logbook for example) of the critical monitoring activities kept on site (temperatures, chlorine levels, etc.)?		−2	
27 *	Random checks of water at least every 6 months		−3	
28	In the control book (if any), there are no abnormal results		−2	
29 *	No *Legionella* detected the last six months (at a concentration of more than 1000 CFU/L)		−3	
**ANNEX ΙΙ: Recordings**				
30 *	Cold water is maintained at temperatures below 25 °C		−3	
31	The cold-water temperature in taps is <25 °C after 2 min flushing		−2	
32	The hot water temperature is >50 °C after 1 min flow		−2	
33 *	The difference in temperature between 2 successive measurements of hot water is NOT > 10 °C/1 min		−3	
34	Hot water store and circulation temp <60 °C		−2	
35	There is NO thermal stratification of the water inside the heaters and storage water		−1	
36	The water temperature is >60 °C when exiting the heating unit and >50 °C when returning to it		−2	
37	Is the pH maintained at 7.2–7.8?		−2	
38 *	There is continuous treatment with chlorine at 0.2–0.5 mg/L?		−3	
39	There is no taste and odor problem		−1	

Inspection results: (A) satisfactory result (0 to 7 points, <10% of the total negative score, no critical violation); (B) relatively satisfactory result (–8 to 14 points, 11–20% of the total negative score, or a critical violation); (C) unsatisfactory result (more than 14 points, >20% of the total negative score).
